# Trastuzumab: a cardiologist’s perspective

**DOI:** 10.3747/co.v15i3.300

**Published:** 2008-06

**Authors:** Marc André Côté

**Affiliations:** Laval University, Centre des maladies du sein Deschênes–Fabia, CHAUQ, Quebec City, Québec

A review of the randomized clinical trials of adjuvant trastuzumab in human epidermal growth factor receptor (her2)–positive breast cancer demonstrates the great benefit of this monoclonal antibody whose potential cardiotoxicity is a fascinating problem for the cardiologist 1. The direct cardiotoxicity of trastuzumab, a relatively new cardiomyopathy, is actually quite limited in the absence of prior anthracycline exposure. Concurrent blockage of the her2 cardiac receptor is believed to make repair of myocardial damage more difficult 2,3, but this effect varies substantially from patient to patient. It is unpredictable, it does not appear to be dose-dependent, and it is reversible. About 80% of patients receiving trastuzumab as adjuvant treatment will maintain a normal left ventricular ejection fraction (lvef), and therefore questions naturally arise about how otherwise to monitor for potential cardiotoxicity.

Cardiac injury can occur in the absence of systolic dysfunction. The lvef measurement can vary from centre to centre (different methods could be used) and even throughout the day by up to 10% in individual patients—a level of fall that could result in treatment being inappropriately suspended. To complicate matters further, the definition of cardiotoxicity as measured by lvef can vary substantially among clinical studies, as can stoppage rules 4. Fortunately, the risk of severe cardiotoxicity remains rare (0.6%–4%), and if such a complication occurs, cessation of trastuzumab therapy can often be followed by contractile improvement given conventional treatment for heart failure. The demonstrable recovery of lvef can even allow for reintroduction of trastuzumab if the cancer prognosis demands it.

The management of an asymptomatic drop of lvef is difficult to define. In 14.2% of patients treated with trastuzumab (as in the B-31 N 9831 trial), an asymptomatic lvef drop triggered stoppage of treatment. In the hera (Herceptin^a^ Adjuvant) trial, in which trastuzumab was given sequentially, and in the subgroup without anthracycline exposure of the Breast Cancer International Research Group (bcirg) 006 trial, a significantly lesser drop in lvef was observed 6. However, from a cardiologist’s viewpoint, a drop in lvef—reversible and symptomatic or not—is always an issue, because it implies an effect on cardiac contractility and actual intracellular damage (and therefore risk of cell death). Furthermore, previous damage caused by anthracyclines is often permanent and significant; the B 31-N9831 showed that 6% of patients who had undergone previous adjuvant anthracycline treatment were unable to begin trastuzumab because of a baseline lvef that was too low 7.

The reversible effect of the decrease in lvef often observed with trastuzumab is disputable. Although it is true that most patients improve once treatment is stopped, many patients need specific cardiac care to achieve that improvement, and many do not regain their initial lvef value.

When encountering a significant asymptomatic drop in lvef, and after appropriate stoppage of trastuzumab 6, some physicians, faced with lack of randomized data on the subject, will wait for spontaneous improvement without a specific cardiac intervention. Others will recommend interventions such as angiotensin converting-enzyme inhibitors in a somewhat accelerated mode, as in any other cardiomyopathy or situation of cardioprotection 6. An ongoing dialogue between the cardiologist and the oncologist is then important to maximize care of the patient!

Now that the benefits of trastuzumab are well established, it is tempting to use this agent to treat patients with prior cardiac problems. However, the effect of trastuzumab on those patients (typically excluded from the defining clinical studies) remains unclear. Accordingly, such patients should be very closely monitored.

The treating physicians must take the lack of knowledge concerning the long-term effects of trastuzumab into consideration, even more so when anthracyclines are part of the equation. Younger patients undergoing trastuzumab adjuvant therapy are obviously at risk from other cardiac stresses in later life and will need cardiac reserve, but it is reassuring that the B 31 and hera studies showed a stable rate of severe cardiotoxicity at 3 years of follow-up.

Given the impact of trastuzumab, cardiologists could rightly question why an alternative to prior anthracycline treatment could not be offered, especially to patients who already have cardiac disease. Is the use of anthracyclines as adjuvant therapy in her2-positive breast cancer still justified? The much-awaited results of bcirg 006 could help to answer that crucial question.

## Footnotes

a (Genentech, San Francisco, CA, U.S.A.)
